# The Role of Brain-Derived Neurotrophic Factor in Irritable Bowel Syndrome

**DOI:** 10.3389/fpsyt.2020.531385

**Published:** 2021-01-14

**Authors:** Thomas Jan Konturek, Cristina Martinez, Beate Niesler, Ivo van der Voort, Hubert Mönnikes, Andreas Stengel, Miriam Goebel-Stengel

**Affiliations:** ^1^Division of Gastroenterology, Loyola University Medical Center, Stritch School of Medicine, Maywood, IL, United States; ^2^Department of Internal Medicine, Institute of Neurogastroenterology, Martin Luther Hospital, Johannesstift Diakonie, Berlin, Germany; ^3^Lleida Institute for Biomedical Research Dr. Pifarré Foundation (IRBLleida), Lleida, Spain; ^4^Department of Human Molecular Genetics, University Hospital Heidelberg, Heidelberg, Germany; ^5^nCounter Core Facility Heidelberg, Institute of Human Genetics, Heidelberg, Germany; ^6^Department of Internal Medicine and Gastroenterology, Berlin Jewish Hospital, Berlin, Germany; ^7^Department of Psychosomatic Medicine, University Hospital Tübingen, Tübingen, Germany; ^8^Department of Internal Medicine and Gastroenterology, Helios Clinic Rottweil, Rottweil, Germany

**Keywords:** comorbidities, colonic biopsy, hypersensitivity, IBS subgroup, symptom severity

## Abstract

Several studies have implied a role of brain-derived neurotrophic factor (BDNF) in abdominal pain modulation in irritable bowel syndrome (IBS). The aim of this study was to establish BDNF protein expression in human colonic biopsies and to show variation in IBS compared to controls. BDNF protein and mRNA levels were correlated with IBS symptom severity based on the IBS-symptom severity score (IBS-SSS). Biopsies from the descending colon and IBS-SSS were obtained from 10 controls and 20 IBS patients. Total protein of biopsies was extracted and assessed by ELISA and Western Blot. Total mRNA was extracted and gene expression measured by nCounter analysis. In IBS patients, symptom severity scores ranged from 124 to 486 (mean ± sem: 314.2 ± 21.2, >300 represents severe IBS) while controls ranged from 0 to 72 (mean ± sem: 27.7 ± 9.0, <75 represents healthy subjects, *p* < 0.001). IBS patients reported significantly more food malabsorption, former abdominal surgery and psychiatric comorbidities. BDNF protein was present in all samples and did not differ between IBS and controls or sex. Subgroup analysis showed that female IBS patients expressed significantly more BDNF mRNA compared to male patients (*p* < 0.05) and male IBS-D patients had higher IBS symptom severity scores and lower BDNF mRNA and protein levels compared to male controls (*p* < 0.05). Scatter plot showed a significant negative correlation between IBS-SSS and BDNF mRNA levels in the cohort of male IBS-D patients and their male controls (*p* < 0.05). We detected a high proportion of gastrointestinal surgery in IBS patients and confirmed food intolerances and psychiatric diseases as common comorbidities. Although in a small sample, we demonstrated that BDNF is detectable in human descending colon, with higher BDNF mRNA levels in female IBS patients compared to males and lower mRNA and protein levels in male IBS-D patients compared to male controls. Further research should be directed toward subgroups of IBS since their etiologies might be different.

## Introduction

Irritable bowel syndrome (IBS) is a debilitating (but not life-threatening) disorder of brain-gut interaction characterized by abdominal pain and dysfunctional bowel habits based on the Rome IV criteria as the latest worldwide standard for the diagnosis of IBS (1).

The pathophysiology of IBS is based on a multifactorial and bidirectional dysfunction of the brain-gut-axis including genetics and epigenetics, visceral hypersensitivity, changes in the synthesis and release of neuropeptides and proinflammatory cytokines and altered gastrointestinal motility as well as psychosomatic predisposition (2).

The brain-derived neurotrophic factor (BDNF) belongs to the family of nerve growth factors (NGF). Through interaction with the tyrosine receptor kinase B (TrkB) (3) BDNF promotes the survival and differentiation of brain neurons, and participates in the modification of neurotransmission and synaptic plasticity of the central and peripheral nervous systems (4). Dysfunctions in epigenetic control, transport or signal cascades of BDNF were discussed on an emergence of various neurological and psychiatric diseases (5). There is also ample evidence of an important role played by BDNF in visceral pain and hypersensitivity conditions (6–11).

Studies in the murine colon showed that BDNF mRNA was expressed in epithelial cells and neurons of the myenteric plexus, and that BDNF levels in the colon were higher than in the brain (12, 13). Similar studies in rats showed that BDNF can be isolated in the distal colonic mucosa. In humans, BDNF has so far been established in the gastric corpus (14), dorsal root ganglia (15, 16), in enteric ganglion cells (17) and in blood (18). Four studies confirmed the presence of BDNF protein in human colonic mucosa of the rectosigmoid junction (11, 19–21).

There is growing evidence of the effects of BDNF on intestinal activity (6–9, 20, 22). Studies in human subjects showed that treatment with recombinant BDNF for several diseases was accompanied by changes in bowel activity. Patients reported a dose-dependent increase in stool frequency and changes in stool consistency, the related mechanism being unclear. In another study, administration of recombinant BDNF in healthy subjects showed an increase in total as well as proximal colonic transit time (23).

Four studies have specifically examined the involvement of BDNF in IBS and its correlation with symptom severity (11, 19–21). Recently, hypermethylated *BDNF* gene, an epigenetic modification, was described in human monocytes and sigmoid colon of IBS patients and was associated with early life stress and psychiatric as well as somatic symptoms (24). However, Videlock et al. performed gene microarray analysis in sigmoid biopsies of subtype-balanced IBS patients and found a multitude of differentially expressed genes, but not BDNF, and only in IBS-C vs. controls (25).

The aim of this study is to demonstrate that BDNF mRNA and protein are detectable in human colonic biopsies and to determine if these correlate with symptom predominance and severity in IBS.

## Materials and Methods

### Study Location

The study was performed with patients with IBS and healthy control subjects at the Institute for Neurogastroenterology at Martin Luther Hospital, a teaching hospital of Charité-Universitätsmedizin in Berlin, Germany between 2011 and 2014. Molecular analysis was carried out at the research institution Charité-Universitätsmedizin Berlin, Campus Virchow Klinikum and Department of Human Molecular Genetics at University of Heidelberg.

### Ethics Commission and Patient Selection

All experimental protocols for the human study were approved by the Clinical Ethical Committee of Charité Universitätsmedizin Berlin (ethical approval number EA1/108/11). All study subjects gave their informed written consent prior to enrollment. All methods used in the human study were carried out in accordance with the approved guidelines and according to standard procedures.

Healthy controls: Healthy subjects were recruited within the framework of preventive colonoscopy for colorectal cancer screening. They had no history of IBS.

IBS patients were newly diagnosed and subclassified according to the ROME III criteria on the basis of the predominant symptom and stool pattern (26). In addition, each subject was screened for organic diseases by medical history taking, physical examination, detailed blood and stool analysis and endoscopy.

Participants were age-matched but not gender-matched. A total of 30 participants were eligible to participate in the study.

### Inclusion and Exclusion Criteria

The following inclusion criteria applied to all study participants: age between 18 and 65 years; body mass index (BMI) between 20 and 25 kg/m^2^; good general condition.

The following criteria led to exclusion of study participants: unstable body weight (weight fluctuation of more than 3 kg within the last month, or weight fluctuation of more than 10 kg in the last 6 months prior to the study); alcohol consumption (>1 alcoholic drink per day); irregular nicotine consumption; pregnancy; use of psychotropic drugs within the last 3 months before the examination; inflammatory bowel disease (IBD); celiac disease; history of malignant tumors and abnormal laboratory values. Laboratory work-up in all study participants included infection parameters with CRP and blood count, INR, electrolytes including sodium and potassium, liver enzymes, kidney function and thyroid hormone TSH. Normal range was based on in-house laboratory standards. Except for sodium, a deviation of >10% (lower or higher) was considered abnormal. For sodium only a deviation of 5% was tolerated. Subjects that displayed parameters outside the normal range were advised to see their general practitioner and could interview again.

### Endoscopy and Material Extraction

For every study subject a complete ileocolonoscopy (CF series, Olympus, Japan) was performed by an experienced gastroenterologist including stepwise biopsies from all sections of the ileocolon for routine histology to rule out pathologies. During withdrawal of the endoscope, five biopsy specimens were removed from the descending colon of each study participant, ~40 to 50 cm above the anocutaneous line. Four colonic biopsy specimens were placed on dry ice and then stored at −80°C, while one biopsy specimen was fixed in formaldehyde at room temperature.

### Questionnaire – IBS Symptom Severity Score (IBS-SSS)

All subjects were asked to answer part 1 of the IBS symptom severity score (IBS-SSS) (27). The questionnaire is meant to register complaint levels related to gastrointestinal symptoms in the form of four questions: (1) Do you suffer from abdominal pain? (2) Do you currently suffer from abdominal distention? (3) How satisfied are you with your bowel habits? (4) Please indicate on the line (visual analog scale) below how much your IBS is affecting or interfering with your life in general? A total of 500 points can be reached. A score of up to 75 points is considered as control, 75–175 points as mild IBS, 175–300 as moderate IBS, and more than 300 as severe IBS.

### RNA Isolation and Quality Assessment

Total RNA was isolated from IBS and control samples. After disruption in TRIzol, the resulting aqueous phase was cleaned-up by the RNAqueous-Micro Total RNA isolation kit (AM1931, Thermo Fisher Scientific) according to the manufacturer's instructions. Quantity and quality of RNA were assessed by Agilent 2100 Bioanalyzer (Agilent Technologies, Waldbronn, Germany) taking into account the RNA integrity number (RIN) value with a cut off of RIN below 5. Samples were stored at −80°C until expression analysis.

### nCounter Analysis

Expression analysis was performed from 100 ng total RNA using the nCounter system Gene 1 (NanoString Technologies, Seattle, USA). A customized codeset comprising 48 target genes including *BDNF* and 7 reference genes was hybridized as recommended by the manufacturer. Background correction and normalization of data was performed using the NanoString software nSolver 3.0 (NanoString Technologies). Stably expressed reference genes were chosen for normalization based on the geNorm method, a popular algorithm to determine the most stable reference genes from a set of tested candidate reference genes in a given sample panel. This algorithm calculates a gene expression normalization factor for each sample based on the geometric mean of a user-defined number of reference genes. The underlying principles and formulas are described in (28). Following this, the selected reference genes were *GAPDH, RPS17, TBP* and *UBC*.

### Gel Electrophoresis and Western Blot Analysis

Ten milliliters of phosphate buffer saline solution (PBS) without calcium and magnesium (PAA Laboratories, Pasching, Austria) was first mixed with half a tablet of proteinase inhibitor cocktail (cOmplete™, Mini, 11836170001, EDTA-free Protease Inhibitor Cocktail, Roche, Mannheim, Germany) and processed in a vortex for 3 s. The finished mixture was distributed in an amount of 400 μl each among lysing matrix tubes (D Matrix, 116913050-CF, MP Biomedicals, California, USA) and placed on ice.

The colonic biopsy specimens stored in the freezer at −80°C were weighed (two biopsies per control subject/patient) with a precision balance (Sartorius, Göttingen, Germany) and then transferred to the prepared matrix tubes, homogenized two times each at 4 m/s for 20 s using a homogenizer (MP Biomedicals, California, USA) and placed on ice for 3 min.

This was followed by centrifugation of the samples at 10 × 1,000 rpm (Eppendorf, Hamburg, Germany) in a cooling chamber at −4°C for 10 min to remove cell debris and nuclei, and subsequent pipetting of the supernatant from the matrix tubes. Final protein concentrations were determined using a BCA protein assay according to the manufacturer's protocol (23225, Pierce Biotechnology, Rockford, IL, USA).

For subsequent sodium dodecyl sulfate - polyacrylamide gel electrophoresis (SDS-PAGE) protein samples were mixed with a gel sample buffer comprising 4% sodium dodecyl sulfate (SDS), 0.05% bromophenol blue solution (w/v), 20% glycerol, 1% mercaptoethanol (v/v) in 0.1 tris(hydroxymethyl)aminomethane (TRIS). The gel samples were immersed for 1 min at 100°C in boiling water, and applied on a 4–12% SDS polyacrylamide gel (Bis-Tris Minigel, NP0321BOX, NuPage; Invitrogen, Carlsbad, CA, USA) with 30 μl of protein per lane. The first lane was filled with 10 μl of a marker (SeeBlue, LC5625, Invitrogen, Carlsbad, CA, USA). A 2-(N-morpholino) ethanesulfonic acid buffer was used as a running buffer. The SDS-PAGE ran for 2 h at 120 V, 300 W, and 350 mA.

After SDS-PAGE, a wet transfer of proteins was carried out by electrophoresis on a nitrocellulose membrane for 1.5 h at room temperature in a TRIS base methanol transfer buffer at pH 8.1–8.4. The membranes were then washed in glass containers using distilled water.

Subsequently, the membranes were stained with Ponceau S staining solution (0.1% Ponceau S and 5% ice acetic acid) to confirm protein transfer. For antibody staining, the membranes were washed twice with TRIS-Tween buffered saline solution (10 mM TRIS, 150 mM NaCl, 0.05% Tween, v/v). This was followed by incubation of the membranes in fat-free milk (Carnation instant skim milk powder, Nestlé, Glendale, CA, USA) for 30 min at room temperature. After removal of the milk, the membranes were washed additionally three times for 5 min each with 15 ml of TRIS-Tween buffered saline solution.

Polyclonal anti-BDNF antibody (ab 72439, Abcam, Cambridge, UK) or a polyclonal antibody against the housekeeping protein β-actin (Ab #4967, Cell Signaling Technology Inc., Danvers, MA, USA) were used in a dilution of 1:5,000 and 1:1,000, respectively, by means of TRIS-Tween buffered saline solution. Incubation took place for 60 min at room temperature on a shaker. This step was followed by a 4-fold washing process with TRIS-Tween buffered saline solution until the secondary antibody was used (alkaline phosphatase conjugated anti-rabbit IgG, S373B 30687401, dilution 1:2,000, Promega, Madison, WI, USA). This was followed by further washing with TRIS-Tween buffered saline solution and subsequent color development in an alkaline phosphatase buffer (100 mM TRIS, 100 mM sodium chloride and 5 mM magnesium chloride, pH 9.5) according to the manufacturer's protocol. For initiation of the 5-min color reaction in the dark, 5 ml of the alkaline phosphatase buffer were added to each membrane, and the two substances nitro-blue tetrazolium (NBT, 0.3%, N6495, Thermo Scientific, Rockford, IL, USA) and 5-bromo-4-chloro-3-indoxyl phosphate (BCIP, 0.15%, 34040, Thermo Scientific, Rockford, IL, USA) were added.

### ELISA

For quantitative determination of the concentration of BDNF protein in colonic biopsies, a commercial BDNF Human ELISA (enzyme-linked immunosorbent assay) Kit (ab99978, Abcam, Cambridge, MA, USA) was used that was based on mouse monoclonal IgG2A antibodies.

The minimum detectable amount of BDNF was 80 pg/ml. Each sample was analyzed in duplicate. The ELISA was run according to the manufacturer's protocol.

### Statistics

Since this was a pilot study, no power analysis was included.

Data are presented as mean ± sem; alternatively, data are indicated as total number and percentage values. Normality was assessed using the Kolmogorow-Smirnov test. Differences were assessed using χ^2^-tests, *t*-tests or the Mann-Whitney-U test depending on the distribution of the data.

Molecular data are presented as mean ± sem. Normality was assessed using the Kolmogorow-Smirnov test. Differences were assessed using *t*-tests or the Mann-Whitney-U test depending on the distribution of the data.

For statistical analysis of nCounter data two-tailed Mann-Whitney U test was used with GraphPad Prism 5.0 (Graph Pad Software, La Jolla, CA, USA). Data are summarized by mean ± standard deviation (SD) or median (range), unless otherwise stated. *P*-values < 0.05 were considered significant.

## Results

### Study Population

Characteristics of the study population are outlined in Table 1. The IBS group comprised 20 patients (14 female, six male) while 10 healthy subjects participated (two female, eight male). The mean age in the IBS and control group was 55.6 and 49.5 years, respectively. No significant differences were noted in ethnicity (most subjects were Caucasian) and in socioeconomic status with most subjects having a university entrance diploma. It is to note that in the IBS group more incomplete data sets were obtained with regards to partnership, children, level of education and employment status.

**Table 1:** Demographic and socioeconomic characteristics, comorbidities, and medication of study patients.

**Parameter**	**Group**		
	**Control (*n* = 10, ♀ = 2, ♂ = 8)**	**IBS (*n* = 20, ♀ = 14, ♂ = 6)**	***p***
**Demographic characteristics**			
Age (years)	55.6 ± 3.0 (38–65)	49.5 ± 3.8 (20–74)	0.301
**Ethnicity**			
Caucasian	10 (100%)	19 (95%)	0.719
Mediterranean	0 (0%)	1 (5%)	
**Socioeconomic characteristics**			
Living in a partnership (yes/no)	6/4	10/5 (5 missing data)	0.932
Children (yes/no)	5/5	11/4 (5 missing data)	0.444
Level of Education		5 missing data	0.870
- University entrance diploma	6 (60%)	9 (60%)	
- Secondary education certificate	2 (20%)	2 (13%)	
- Basic school qualification	2 (20%)	4 (27%)	
- Without school-leaving qualification	0 (0%)	0 (0%)	
Currently employed (yes/no)	5/5	8/7 (5 missing data)	0.806
**Comorbidities**			
Gastrointestinal	6 (60%)	13 (68%, 1 missing data)	0.996
- Reflux esophagitis	2 (20%)	2 (11%)	
- Diaphragmatic hernia	0 (0%)	1 (5%)	
- Gastritis	2 (20%)	4 (21%)	
- Duodenal ulcer	0 (0%)	1 (5%)	
- Diverticular disease	2 (20%)	3 (16%)	
- Hämorrhoids	0 (0%)	2 (11%)	
Malabsorption	0 (0%)	17 (89%, 1 missing data)	**<0.001**
- Fructose malabsorption	0 (0%)	10 (53%)	
- Lactose intolerance	0 (0%)	4 (21%)	
- Histamine intolerance	0 (0%)	1 (5%)	
- Bile acid malabsorption	0 (0%)	1 (5%)	
- Vitamin B12 deficiency	0 (0%)	1 (5%)	
IBS-associated	0 (0%)	8 (42%, 1 missing data)	**0.048**
Abdominal pain syndrome	0 (0%)	1 (5%)	
Somatoform disorder	0 (0%)	1 (5%)	
Anxiety disorder	0 (0%)	1 (5%)	
Dysthymia	0 (0%)	1 (5%)	
Insomnia	0 (0%)	1 (5%)	
Migraine	0 (0%)	1 (5%)	
Chronic pain syndrome	0 (0%)	1 (5%)	
Fibromyalgia	0 (0%)	1 (5%)	
Metabolic	3 (30%)	12 (63%, 1 missing data)	0.191
- Hyperuricemia	1 (10%)	0 (0%)	
- Diabetes mellitus Type 2	0 (0%)	1 (5%)	
- Fatty liver disease	0 (0%)	3 (16%)	
- Hypothyroidism	0 (0%)	3 (16%)	
- Hyperlipoproteinemia	1 (10%)	2 (11%)	
- Nephrolithiasis	1 (10%)	1 (5%)	
- Cholecystolithiasis	0 (0%)	1 (5%)	
- Pancreas divisum	0 (0%)	1 (5%)	
Cardiovascular	3 (30%)	6 (32%, 1 missing data)	0.738
- Arterial hypertension	2 (20%)	3 (16%)	
- Arteriosclerosis	0 (0%)	1 (5%)	
- Past stroke	0 (0%)	1 (5%)	
- Past embolism	1 (10%)	1 (5%)	
Other	3 (30%)	7 (37%, 1 missing data)	0.966
- Gynecological disorder	0 (0%)	2 (11%)	
- Benign prostate hyperplasia	2 (20%)	0 (0%)	
- Degenerative orthopedic condition (e.g., arthrosis, disc etc.)	1 (10%)	5 (26%)	
Former surgery	0 (0%)	8 (42%, 1 missing data)	**0.048**
- Cholecystectomy	0 (0%)	4 (21%)	
- Appendectomy	0 (0%)	3 (16%)	
- Bowel surgery	0 (0%)	2 (11%)	
- Hernioplastic	0 (0%)	1 (5%)	
- Hysterectomy	0 (0%)	5 (26%)	
- Ovarectomy	0 (0%)	3 (16%)	
- Tonsillectomy	0 (0%)	1 (5%)	
**Medication**			
Medication use	3 (30%)	12 (63%, 1 missing data)	0.191
- Proton pump inhibitors	2 (20%)	2 (11%)	
- Antihypertensives	1 (10%)	4 (21%)	
- Vitamin B12	0 (0%)	1 (5%)	
- Thyroid supplementation	0 (0%)	3 (16%)	
- Gynecological hormone substitution	0 (0%)	1 (5%)	
- Statins	0 (0%)	2 (11%)	
- Antidiabetics	0 (0%)	1 (5%)	
- Sleep aids	0 (0%)	1 (5%)	
- Selective serotonine reuptake inhibitors	0 (0%)	1 (5%)	
- Tricyclic antidepressants	0 (0%)	2 (11%)	
- Neuroleptics	0 (0%)	1 (5%)	
- Budenoside	0 (0%)	1 (5%)	
- Mesalazine	0 (0%)	1 (5%)	
- Prostate medication	1 (10%)	0 (0%)	
- Laxatives	0 (0%)	1 (5%)	
- Antispasmodics	0 (0%)	1 (5%)	
- Antidiarrheals	0 (0%)	1 (5%)	

*Data are presented as mean ± sem, the range is indicated in parentheses; alternatively, data are indicated as total number and percentage values in parentheses. Normality was assessed using the Kolmogorow-Smirnov test. Differences were assessed using χ^2^-tests, t-tests or the Mann-Whitney-U test depending on the distribution of the data. Significant differences are displayed in bold. IBS, irritable bowel syndrome*.

Comparing comorbidities, 89% of subjects in the IBS group were suffering from an intestinal malabsorption compared to 0% in the control group (*p* < 0.001) with fructose malabsorption and lactose intolerance being most prevalent. Somatoform and psychiatric disorders which have been associated with IBS (29) were of a wide spectrum and could be found in 42% (*p* = 0.048) of subjects in the IBS group compared to 0% in the control group.

Diagnosis of gastritis and peptic ulcer disease (Table 1) was based on patient charts and history. Two controls reported nonspecific chronic gastritis. Of IBS patients, 2 had a history of Helicobacter pylori (H.pylori) positive antrum or corpus gastritis, respectively, while two had H. pylori negative antrum gastritis and one had a history of healing duodenal ulcer. Of those five patients, only one (H. pylori negative antrum gastritis) was on proton pump inhibitor medication at the time of study. Neither patients nor controls underwent diagnostics for functional dyspepsia.

There was also a significantly higher amount of prior abdominal surgeries noted in the IBS group (42 vs. 0% in control group, *p* = 0.048). Hysterectomy and cholecystectomy were the most frequent surgeries in the IBS group with 26 and 21%, respectively. Three women had undergone oophorectomies but were past menopause and not taking any hormonal supplements.

Overall, IBS patients tended to take more medications than control subjects (*p* = 0.191).

### BDNF Is Detectable in Human Descending Colonic Tissue With Similar Levels in IBS Patients and Healthy Controls

**Figure 1:**
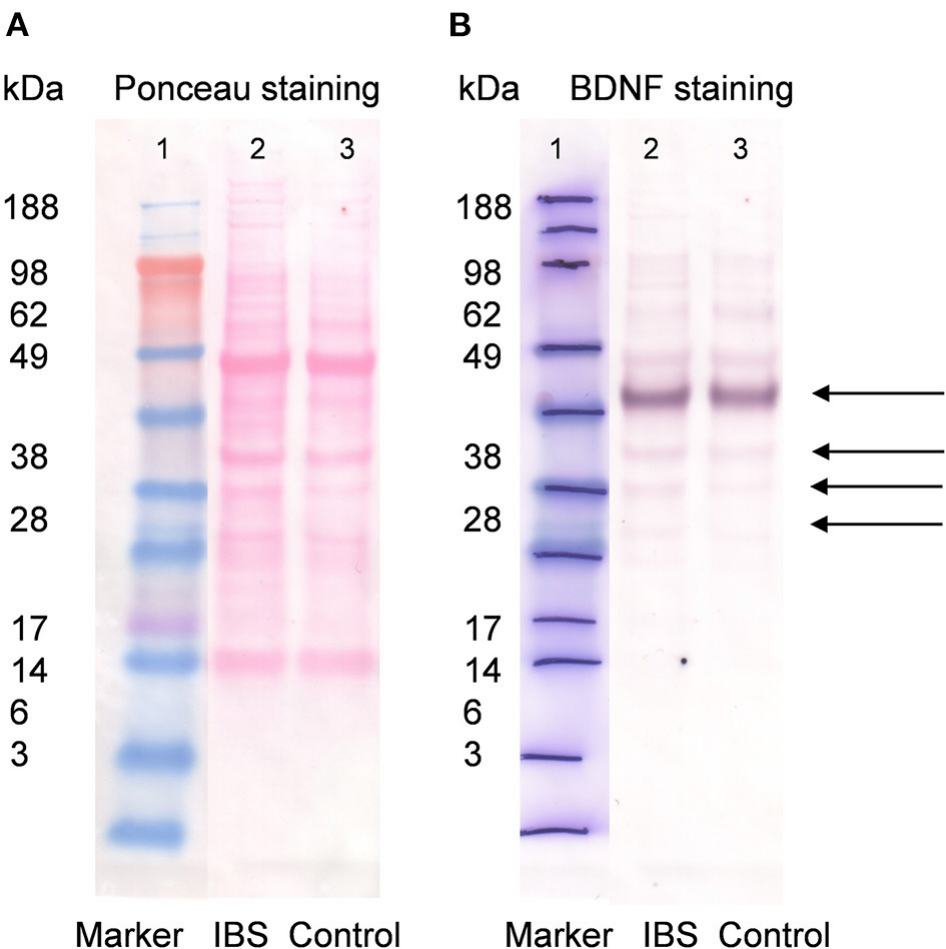
Western blot for BDNF (1:5,000) in IBS patients and control subjects. Lane 1 contains the molecular weight standards. Lane 2 contains colonic wall protein obtained by deep tissue biopsy from mucosa/submucosa in patients with IBS (14 female and six male, pooled sample) and lane 3 colonic wall protein from control subjects (two female and eight male, pooled sample, **A**). Same blot after washout of ponceau staining solution and application of primary anti-BDNF antibody (1:5,000) and secondary antibody goat anti-rabbit AP (1:2,000). Lane 1 contains molecular standards highlighted with pen. Lane 2 and 3 show detection of multiple bands with similar intensity for patients with IBS and healthy controls, respectively. For BDNF (UniProt P23560) multiple Western Blot bands are possible and expected. The strongest band was found between 49 and 62 kDa most likely representing glycosylated prepro-/pro BDNF dimer. Weaker bands were detected at ~ 45, 38, and ~ 30 kDa likely related to prepro-/pro BDNF **(B)**.

**Figure 2:**
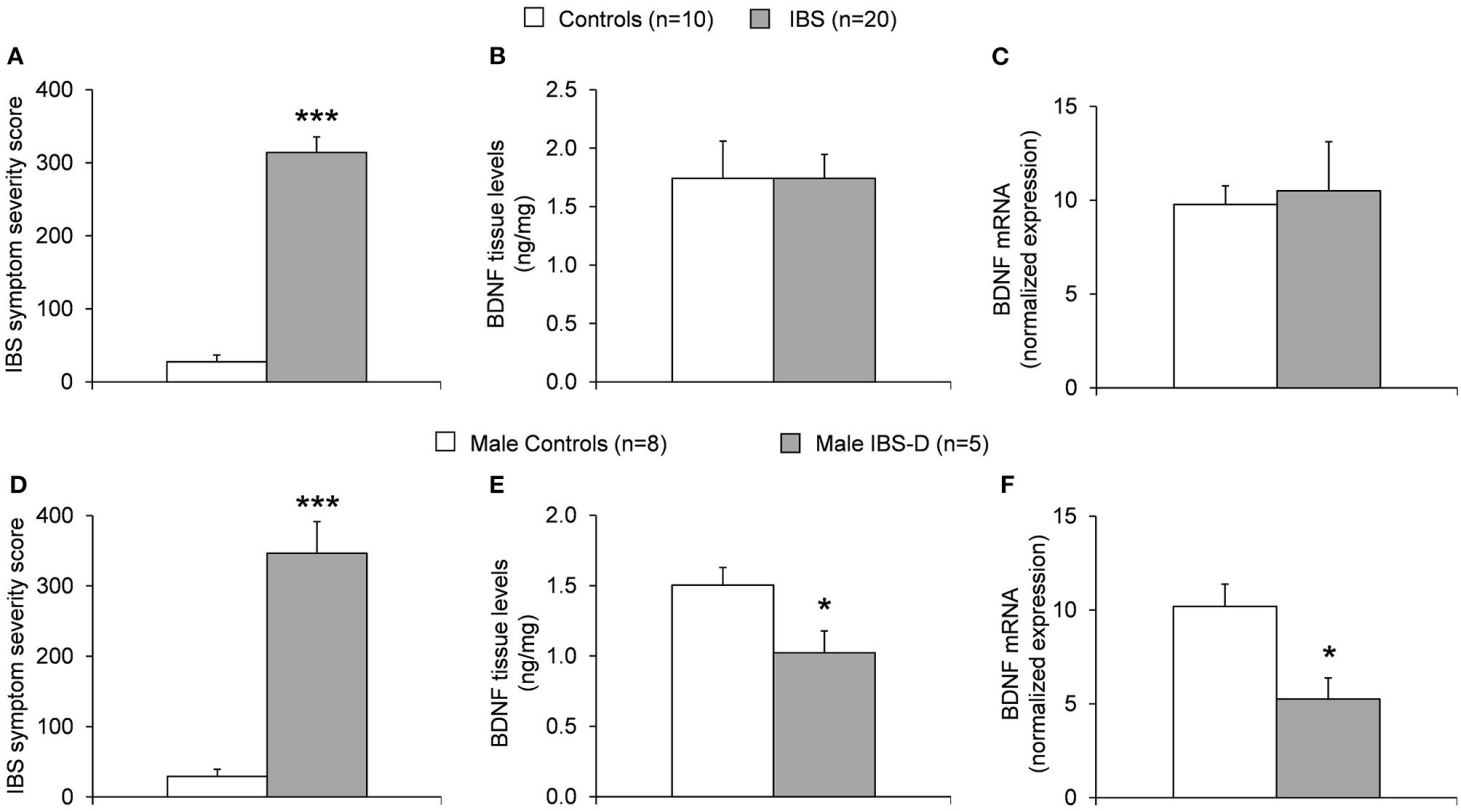
**(A)** IBS symptom severity in healthy controls vs. IBS patients. Significantly higher symptom severity scores are notable in the IBS population vs. controls (***p < 0.001). **(B)** Measurement of BDNF protein tissue levels obtained from colonic biopsies of the descending colon by nCounter analysis in healthy controls (*n* = 10, two female and eight male) and IBS patients (*n* = 20, 14 female and six male). No notable difference is detected in BDNF protein levels in healthy controls vs. IBS patients. **(C)** Measurement of BDNF mRNA levels obtained from colonic biopsies of the descending colon by ELISA in healthy controls (*n* = 10) and IBS patients (*n* = 20) with no notable difference detected. **(D)** IBS symptom severity in healthy male controls vs. male IBS-D patients. Significantly higher symptom severity scores are notable in the male IBS-D population vs. male controls (***p < 0.001). **(E)** Measurement of BDNF protein tissue levels obtained from colonic biopsies of the descending colon by nCounter analysis in healthy male controls (*n* = 8) and male IBS-D patients (*n* = 5). BDNF protein levels in male IBS-D patients were significantly lower compared to male healthy controls (*p < 0.05). **(F)** Measurement of BDNF mRNA levels obtained from colonic biopsies of the descending colon by ELISA in healthy male controls (*n* = 8) and male IBS-D patients (*n* = 5). BDNF mRNA levels in male IBS-D patients were significantly lower compared to male healthy controls (*p < 0.05).

Western blot analysis of human colonic biopsies containing mucosa and submucosa of the descending colon stained with anti-BDNF antibody indicated multiple prominent bands (Figure 1). The presence of different molecular weight forms of BDNF has been reported in prior studies using cultured neuronal and non-neuronal cells (30, 31) and represents differently glycosylated and glycosulfated forms of mature BDNF and proBDNF (32). BDNF was further successfully quantified using ELISA confirming the above Western Blot findings (Figures 2B,E, Supplementary Figures 1B,E) and by nCounter analysis (Figure 2C). No notable differences in BDNF protein and BDNF mRNA expression between healthy controls and IBS patients were detected.

### Characterization of IBS in Patient Population

In the IBS group, 14 subjects met criteria for diarrhea-predominant IBS (IBS-D), 2 for constipation-predominant IBS (IBS-C), 3 for IBS with mixed bowel habits (IBS-M) and 1 for unsubtyped IBS. Mean duration of disease in the IBS group was 6.1 ± 1.5 (1–22) years.

Mean symptom severity was significantly higher in the IBS group as compared to controls (314.2 ± 20.7 vs. 27.7 ± 8.5, *p* < 0.001, Figure 2A, Supplementary Figure 1A). No differences in symptom severity were noted in men and women both within the IBS group and the control group (Table 2).

**Table 2:** Characterization of irritable bowel syndrome in patient population.

**Parameter**	**Group**		
	**Control (*n* = 10, ♀ = 2, ♂ = 8)**	**IBS (*n* = 20, ♀ = 14, ♂ = 6)**	***p***
**IBS subgroup**			
Diarrhea	n.a.	14	n.a.
Constipation	n.a.	2	
Mixed	n.a.	3	
Unsubtyped	n.a.	1	
**Duration of disease**			
Duration (years)	n.a.	6.1 ± 1.5 (1–22)	n.a.
**Severity**			
IBS Symptom Severity Score	27.7 ± 9.0 (0–72)	314.2 ± 21.2 (124–486)	**<0.001**
IBS-SSS grade			**<0.001**
0	10	0 (0%)	
1	0	2 (10%)	
2	0	8 (40%)	
3	0	10 (50%)	
4	0	0 (0%)	

*Data are presented as mean ± sem, the range is indicated in parentheses; alternatively, data are indicated as total number and percentage values in parentheses. Normality was assessed using the Kolmogorow-Smirnov test. Differences were assessed using χ^2^-tests or t-tests. Significant differences are displayed in bold. IBS, irritable bowel syndrome; IBS-SSS, IBS Symptom Severity Score; n.a., not applicable*.

Based on the IBS-SSS, most subjects in the IBS group were classified as either having severity grade 2 (40%) or severity grade 3 (50%).

### Women With IBS Show Higher BDNF Levels Than Men With IBS

Female IBS patients had significantly more BDNF mRNA expression compared to male IBS patients (13.2 ± 3.6 vs. 4.7 ± 1.1, *p* < 0.05).

BDNF protein tissue levels in women with IBS compared to men with IBS were not significantly different (2.0 ± 0.3 ng/mg vs. 1.2 ± 0.2; *p* = 0.076) when analyzed with ELISA (Table 3). Due to limited group size no comparison of female and male controls was performed.

**Table 3:** IBS symptom severity scores and BDNF levels according to sex.

**Parameter**	**Group**					
	**Control (*****n*** **= 10)**			**IBS (*****n*** **= 20)**		
	**Women (*n* = 2)**	**Men (*n* = 8)**	***p***	**Women (*n* = 14)**	**Men (*n* = 6)**	***p***
IBS-SSS	21.5 ± 19.5 (2–41)	29.3 ± 10.1 (0–72)	n.c.	293.7 ± 23.0 (124–439)	361.8 ± 39.8 (220–486)	0.133
BDNF mRNA (arbitrary unit)	8.1 ± 1.4 (6.7–9.4)	10.2 ± 1.2 (7.5–16.8)	n.c.	13.2 ± 3.6 (1.4–48.1)	4.7 ± 1.1 (1.9–8.6)	**0.048**
BDNF protein (ng/mg)	2.7 ± 1.6 (1.1–4.3)	1.5 ± 0.1 (0.9–1.9)	n.c.	2.0 ± 0.3 (0.6–3.4)	1.2 ± 0.2 (0.6–1.8)	0.076

*BDNF levels were assessed using nCounter analysis and ELISA and corrected for housekeeping gene expression or total tissue protein, respectively. Data are presented as mean ± sem, the range is indicated in parentheses. Normality was assessed using the Kolmogorow-Smirnov test. Differences were assessed using t-tests or the Mann-Whitney-U test depending on the distribution of the data. Significant differences are displayed in bold. BDNF, brain-derived neurotrophic factor; IBS, irritable bowel syndrome; IBS-SSS, IBS Symptom Severity Score; n.c., not calculated*.

### Men With IBS-D Have Lower BDNF Levels Than Male Controls

Comparing the whole IBS-D group to controls, BDNF protein (1.5 ± 0.2 ng/mg vs. 1.74 ± 0.3; *p* = 0.500) or mRNA (7.0 ± 1.0 ng/ml vs. 1.74 ± 0.3; *p* = 0.07) levels were not different. IBS-SSS was significantly higher with 297.4 ± 21.0 points vs. 27.7 ± 9.0 (mean ± SEM; *p* < 0.0001).

Further dividing the IBS-D subgroup into male and female, five male IBS-D patients were compared to eight male controls. Here, the IBS-SSS was significantly higher in male IBS-D compared to male controls (346.6 ± 45.0 vs. 29.3 ± 10.1, *p* < 0.001). BDNF protein (1.0 ± 0.2 ng/mg vs. 1.5 ± 0.1, *p* < 0.05) and mRNA levels (5.3 ± 1.1 vs. 10.2 ± 1.2, *p* < 0.05) were significantly lower compared to controls (Figures 2D–F, Supplementary Table 1, Supplementary Figures 1D–F).

The female IBS-D subgroup was comprised of eight individuals compared to two female controls. Data are shown for completion in Supplementary Table 1 but were not statistically analyzed.

### BDNF Levels Do Not Differ in IBS Patients With Fructose Malabsorption

Except one, all patients with fructose malabsorption had IBS-D with a sex ratio of seven female and three male. Mean symptom severity was significantly higher in the IBS + fructose malabsorption group compared to IBS without fructose malabsorption (312.1 ± 28.6 vs. 316.2 ± 31.4, *p* = 0.575). In IBS with fructose malabsorption, BDNF protein levels (1.7 ± 0.3 ng/mg vs. 1.8 ± 0.3; *p* = 0.904) and BDNF mRNA levels (10.8 ± 2.8 vs. 10.2 ± 4.4, *p* = 0.918) did not differ from IBS without fructose malabsorption. Data are shown in Supplementary Table 2.

### Correlation of IBS-SSS and BDNF Levels

**Figure 3:**
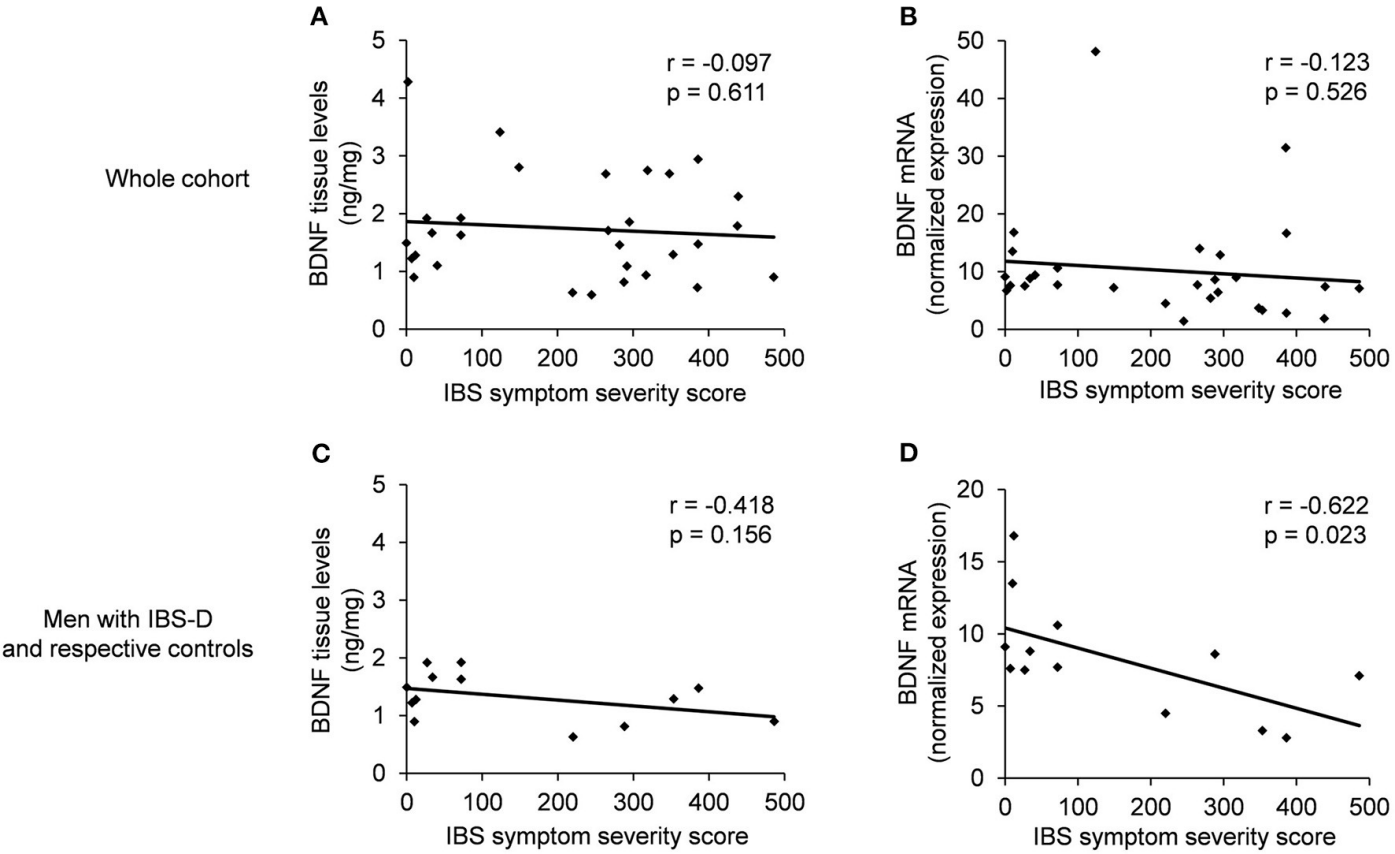
Correlation between gastrointestinal symptom severity vs. BDNF protein tissue levels from the descending colon or BDNF mRNA levels. **(A,B)** shown the whole cohort (10 controls: eight female and two male; 20 IBS: 14 female and six male) and **(C,D)** show the subgroups of male IBS-D patients (*n* = 5) with their male controls (*n* = 8). Scatter plot showing **(A,B)** no significant correlation between IBS-SSS and BDNF protein or mRNA levels in the whole cohort (*r* = −0.097; *p* = 0.611 and *r* = −0.123; *p* = 0.526) and **(C)** no significant correlation between IBS-SSS and BDNF protein tissue levels in male IBS-D patients and respective control cohort (*r* = −0.418; *p* = 0.156) and **(D)** a significant negative correlation between IBS-SSS and BDNF mRNA levels in male IBS-D patients and male controls (*r* = −0.622; *p* = 0.023).

There was no significant correlation between BDNF protein or mRNA levels and the severity of IBS symptoms in the study cohort (*r* = −0.097, *p* = 0.611 and *r* = −0.123, *p* = 0.526, respectively, Figures 3A,B).

In male IBS-D patients (*n* = 5) and their male controls (*n* = 8), BDNF protein did not significantly correlate (*p* = 0.156) but mRNA levels showed a significant negative correlation (*p* = 0.023) with IBS symptom score (Figures 3C,D).

## Discussion

Over a century ago Sir William Osler introduced the term mucous colitis and described a disorder of mucorrhea and abdominal colic with a high incidence in patients presenting with psychopathology (33). Over the decades, IBS has been diagnosed by means of exclusion and at the present time it still remains a diagnostic challenge. The pathophysiology of this disorder remains unclear and may be different depending on the subtype. Nonetheless, a disturbed physiological interplay between colon motility, hormones and transmitters, visceral hyperalgesia and psychopathology results in this heterogeneous symptom complex (2).

BDNF is suspected to be involved in the regulation of colonic motility as well as in the control of visceral hyperalgesia (6–10, 23). The aim of our study was to detect and quantify BDNF levels in human biopsies from the descending colon in patients with IBS and healthy controls and correlate them with IBS symptoms and severity.

We were able to demonstrate that BDNF is present in human descending colon biopsies obtained by endoscopy. Similar observations, however in biopsies from the rectosigmoid, were made by Yu et al. (19), Wang et al. (20) (affiliated groups) and Zhang et al. (21). Only Wang et al. performed Western Blot in human rectosigmoid biopsies for BDNF showing a cut-out band between 20 and 30 kDa (20). Our Western Blot analysis revealed multiple molecular weight forms of BDNF. Interestingly, the strongest band was found between 49 and 62 kDa with weaker bands at 45, 38, and 30 kDa. The presence of different molecular weight forms of BDNF has been reported in prior studies using cultured neuronal and non-neuronal cells (30, 31). It has been suggested that the variety of bands is due to differently glycosylated and glycosulfated forms of proBDNF and mature BDNF (32). Analysis of adult rat spinal cord revealed that BDNF knockout mice showed a positive band at 55 kDa when stained with BDNF antibody suggestive of a non-specific finding (34). It can be speculated whether the presence of the strongest band between 49 and 62 kDa is also a non-specific finding and that the 45, 38, and 30 kDa bands represent real BDNF protein. Given that the mature form of BDNF monomer is usually found at 14 kDa as suggested by the manufacturer (ab 72439, Abcam, Cambridge, UK), we believe that our western blot findings represent the proBDNF form which is usually found at around 34 kDa likely depending on the degree of glycosylation/glycosulfation. ProBDNF is of importance for proper dimerization, folding as well as targeting of mature BDNF, however, it has been found that this form also elicits its own distinct effects opposing those of mature BDNF (35). It has been shown that proBDNF induces cellular apoptosis (36), whereas the mature form promotes neuroplasticity and cell differentiation (37). On the cellular level, after binding to the TrkB receptor, mature BDNF results in recruitment of proteins that activate three distinct signaling pathways: Ras/MAPK-ERK pathway, PI3-K pathway and PLC pathway (38). So far Fu et al. could demonstrate in a rodent model with bowel obstruction that peripheral up-regulation of BDNF expression may play a critical role in the abnormal hyper-excitability of primary sensory neurons. Intraperitoneal administration of a TrkB inhibitor resulted in blocked hyper-excitability of colon neurons, which was associated with attenuation of referred visceral hypersensitivity (39). More investigations are needed to further evaluate the cellular actions of BDNF in the gut.

To our surprise in contrast to prior studies (11, 19–21), no significant differences in the concentration of BDNF protein and mRNA were found in the IBS cohort compared to controls. It can be speculated whether this is attributed to a different study population. Compared to Zhang et al. (21) we did include patients with carbohydrate malabsorption as well as patients with previous abdominal surgeries. Most of our patients were women while Zhang's study population consisted of more males. Our sample size is also smaller. However, in our opinion the reason for this discrepancy of results may be mainly due to the different location in the colon the biopsies were taken from. In all prior investigations, biopsies were obtained from the rectosigmoid junction whereas our biopsies were taken from the descending colon (~40 to 50 cm above the anocutaneous line). In the physiological state the rectosigmoid colon is responsible for storage and eventually defecation while the more proximal parts of the colon are involved in mixing and propelling of the content and thus securing its optimal contact to the mucosal wall to absorb the water and eventually solidifying fecal contents (40). The different motility pattern of the rectosigmoid colon compared to the rest of the colon is a well-known phenomenon which has been previously attributed to a higher rate of the basic electrical rhythm (BER) probably resulting in occurrence of more intense motility observed in the rectosigmoid colon caused mainly by giant migrating contractions (40, 41). Our results showing no substantial difference between BDNF tissue concentration in healthy subjects and IBS patients suggest that the descending colon and probably parts of the ascending and transverse colon are not or at least to a lesser degree responsible for symptoms occurring in IBS mediated by BDNF. Unfortunately, there is no substantial clinical data on regional differences of visceral sensitivity of the colon and further investigations are needed to elucidate this phenomenon as well as regional BDNF expression, especially with regard to patients with IBS. Furthermore, our heterogeneous IBS group could be the reason why overall no differences in IBS vs. control were found. Analysis of the IBS-D subgroup showed that male IBS-D patients had significantly lower BDNF mRNA and protein levels compared to male controls. This supports the hypothesis that the subforms of IBS have many different etiologies and especially IBS-D may underlie a different pathology. As recently reviewed, NGFs together with mast cells modulate visceral sensitivity, intestinal barrier function, and motility in IBS-D (42). Unfortunately, due to limited subgroup size no further analyses of the other subgroups could be performed and one should keep in mind that the IBS-D subgroup is of small sample size as well. Thus, results should be interpreted with caution. Whether the differences found are exclusive to male IBS-D must be ascertained in future studies with higher numbers of sex-categorized and subgrouped IBS patients.

Our study reveals significantly higher levels of measurable BDNF mRNA in female IBS patients as compared to male IBS patients. A 2:1 female to male predominance of IBS is well known (43, 44) and could be reproduced in our study. Previous studies suggest that regulation of BDNF and its receptors is controlled by estrogen (45, 46). Recently, Ji et al. investigated the opposing roles of estradiol and testosterone on stress-induced visceral hypersensitivity (SIVH) in rats. It could be shown that estradiol injection in intact male rats increased both SIVH and BDNF levels in the spinal cord whereas testosterone injection in female rats attenuated SIVH and decreased BDNF levels. One rodent study looked at colonic BDNF levels after a single inflammatory insult to the distal colon and found strong up-regulation only in rats with concomitant high estrogen levels while the inflammation itself did not induce BDNF changes (47). Both studies support the hypothesis that estrogens seem to be involved in BDNF upregulation.

Our study included a total of 10 controls and 20 patients with IBS. Given the limited study size it is possible that our results could not reach significance. Furthermore, there was a disproportionate gender distribution within the two study groups without gender matching making results prone to bias. The IBS group included 14 women and six men whereas the control group consisted of mostly men (*n* = 10, ♀ = 2, ♂ = 8).

Our study population in many aspects represents typical demographic and epidemiologic findings as well as associated comorbidities of IBS. Interestingly, most of our patients in the IBS group owned a university entrance diploma or a secondary education certificate which supports data that a higher socioeconomic status is associated with IBS (48). We speculate if in a country like Germany with universal health care access, a possible reason for this phenomenon is a higher level of stress perceived by people working in the professional and managerial field.

Malabsorption, in particular fructose malabsorption, has been shown to be associated with IBS (49, 50). Our study population is representative in that regard as 53% of IBS patients had concurrent fructose malabsorption diagnosed prior to evaluation for IBS. No differences in BDNF levels in that subgroup could be detected. Except one, all patients with fructose malabsorption had IBS-D. The exact reason for this association remains unclear, however, possible suggested mechanisms include alteration of the enteric microbiome, changes in intestinal permeability, rapid small bowel transit, and immune responsiveness (51, 52). Education on nutrition in this subgroup of patients may be of benefit for better symptom control (49, 53). Similar associations were found with lactose intolerance and IBS (54).

IBS is further associated with a variety of somatic and psychiatric conditions which is reflected in our IBS study population in 42% of cases compared to 0% in the control group (*p* = 0.048). Recognition and treatment of these comorbidities is pertinent since it has been shown that there is a correlation with enhanced medical help seeking, worse prognosis and higher rates of depression and anxiety (29). These patients often undergo extensive diagnostic workup which may include surgical interventions (48). It needs to be emphasized that all of our IBS patients have a history of abdominal surgery. This association could raise suspicion that IBS is associated with intraabdominal/peritoneal adhesions, however, it could also reflect the poor diagnostic knowledge on IBS in the German healthcare setting leading to false diagnoses and common surgeries such as cholecystectomies or appendectomies.

IBS remains a debilitating disorder of brain-gut interaction. Although in a small sample, our study detected a high proportion of gastrointestinal surgery in IBS patients and confirmed food intolerances and psychiatric diseases as common comorbidities of IBS. We demonstrated that BDNF, a nerve growth factor suspected to be involved in the control of visceral hyperalgesia and colon motility, is detectable in human biopsies of the descending colon with lower levels in the small subgroup of male IBS-D patients suggesting that this subgroup may be different from the others. No difference was found between the whole IBS cohort and controls which could be due to the heterogeneous IBS subgroups. Regional differences in colonic BDNF expression could also account for these findings although this conclusion cannot be drawn from our current data that focused on BDNF expression in the descending colon only. A regional characterization of BDNF expression should thus be subject of future studies. The finding that female IBS patients show higher BDNF levels than male patients supports previous data that sex hormones are involved in the regulation of BDNF. Extrapolating from the current findings, female IBS patients as well as male IBS-D patients should be studied in a larger sample size to further characterize the role of BDNF in the pathophysiology of IBS.

## Data Availability Statement

The datasets generated for this study are available on request to the corresponding author.

## Ethics Statement

The studies involving human participants were reviewed and approved by Clinical Ethical Committee of Charité Universitätsmedizin Berlin (ethical approval number EA1/108/11). The patients/participants provided their written informed consent to participate in this study.

## Author Contributions

TK performed experiments and wrote the manuscript. CM performed experiments. BN performed experiments and edited the manuscript. IV and HM performed experiments. HM provided some resources. AS edited the manuscript and provided some resources. MG-S performed experiments, reviewed the literature, wrote and edited the manuscript, and provided the resources. All authors contributed to the article and approved the submitted version.

## Conflict of Interest

AS is consultant for a & r Berlin, Boehringer-Ingelheim, Takeda and Dr. Willmar Schwabe GmbH. MG-S is consultant for Dr. Willmar Schwabe GmbH and Yakult. The remaining authors declare that the research was conducted in the absence of any commercial or financial relationships that could be construed as a potential conflict of interest.

## Supplementary Material

Supplementary Figure 1 Graphs are based on pooled data shown in Figure 2. The Supplementary Figure additionally shows individual data values. Details and levels of significance are displayed in Figure 2. **(A)** IBS symptom severity in healthy controls vs. IBS patients. **(B)** Measurement of BDNF protein tissue levels obtained from colonic biopsies of the descending colon by ELISA in healthy controls and IBS patients. **(C)** Measurement of BDNF mRNA levels obtained from colonic biopsies of the descending colon by ELISA in healthy controls and IBS patients. **(D)** IBS symptom severity in healthy male controls vs. male IBS-D patients. **(E)** Measurement of BDNF protein tissue levels obtained from colonic biopsies of the descending colon by ELISA in healthy male controls and male IBS-D patients. **(F)** Measurement of BDNF mRNA levels obtained from colonic biopsies of the descending colon by ELISA in healthy male controls and male IBS-D patients.

Supplementary Table 1 IBS symptom severity scores and BDNF levels in IBS-D subgroup.

Supplementary Table 2 IBS-SSS and BDNF levels in IBS patients without fructose malabsorption (IBS – FM) or with (IBS + FM).

## References

[1] Drossman DA Hasler WL. Rome IV-functional gi disorders: disorders of gut-brain interaction. Gastroenterology. (2016) 150:1257–61. 10.1053/j.gastro.2016.03.035 27147121

[2] Enck P Aziz Q Barbara G Farmer AD Fukudo S Mayer EA. Irritable bowel syndrome. Nat Rev Dis Primers. (2016) 2:16014. 10.1038/nrdp.2016.14 27159638 PMC5001845

[3] Lewin GR Barde YA Physiology of the neurotrophins. Annu Rev Neurosci. (1996) 19:289–317. 10.1146/annurev.ne.19.030196.001445 8833445

[4] Leibrock J Lottspeich F Hohn A Hofer M Hengerer B Masiakowski P. Molecular cloning and expression of brain-derived neurotrophic factor. Nature. (1989) 341:149–52. 10.1038/341149a0 2779653

[5] Benarroch EE. Brain-derived neurotrophic factor: regulation, effects, and potential clinical relevance. Neurology. (2015) 84:1693–704. 10.1212/WNL.0000000000001507 25817841

[6] Zhu ZW Friess H Wang L Zimmermann A Buchler MW. Brain-derived neurotrophic factor. (BDNF) is upregulated and associated with pain in chronic pancreatitis. Dig Dis Sci. (2001) 46:1633–9. 10.1023/A:1010684916863 11508661

[7] Obata K Noguchi K. BDNF in sensory neurons and chronic pain. Neurosci Res. (2006) 55:1–10. 10.1016/j.neures.2006.01.005 16516994

[8] Li CQ Xu JM Liu D Zhang JY Dai RP. Brain derived neurotrophic factor (BDNF) contributes to the pain hypersensitivity following surgical incision in the rats. Mol Pain. (2008) 4:27. 10.1186/1744-8069-4-27 18637202 PMC2492846

[9] Yang J Yu Y Yu H Zuo X Liu C Gao L. The role of brain-derived neurotrophic factor in experimental inflammation of mouse gut. Eur J Pain. (2010) 14:574–9. 10.1016/j.ejpain.2009.10.007 19932037

[10] Joo YE. Increased expression of brain-derived neurotrophic factor in irritable bowel syndrome and its correlation with abdominal pain. (Gut 2012;61:685-694). J Neurogastroenterol Motil. (2013) 19:109–11. 10.5056/jnm.2013.19.1.109 23350058 PMC3548116

[11] Qi Q Chen F Zhang W Wang P Li Y Zuo X. Colonic N-methyl-d-aspartate receptor contributes to visceral hypersensitivity in irritable bowel syndrome. J Gastroenterol Hepatol. (2017) 32:828–36. 10.1111/jgh.13588 27575648

[12] Lommatzsch M Braun A Mannsfeldt A Botchkarev VA Botchkareva NV Paus R. Abundant production of brain-derived neurotrophic factor by adult visceral epithelia. Implications for paracrine and target-derived Neurotrophic functions. Am J Pathol. (1999) 155:1183–93. 10.1016/S0002-9440(10)65221-2 10514401 PMC1867012

[13] Lucini C Maruccio L de Girolamo P Vega JA Castaldo L. Localisation of neurotrophin - containing cells in higher vertebrate intestine. Anat Embryol. (2002) 205:135–40. 10.1007/s00429-002-0237-x 12021915

[14] Cheung CKY Lan LL Kyaw M Mak ADP Chan A Chan Y. Up-regulation of transient receptor potential vanilloid (TRPV) and down-regulation of brain-derived neurotrophic factor (BDNF) expression in patients with functional dyspepsia (FD). Neurogastroenterol Motil. (2018) 30, 1–9. 10.1111/nmo.13176 28782273

[15] Yamamoto M Sobue G Yamamoto K Terao S Mitsuma T. Expression of mRNAs for neurotrophic factors (NGF, BDNF, NT-3, and GDNF) and their receptors (p75NGFR, trkA, trkB, and trkC) in the adult human peripheral nervous system and nonneural tissues. Neurochem Res. (1996) 21:929–38. 10.1007/BF02532343 8895847

[16] Pluchino N Cubeddu A Begliuomini S Merlini S Giannini A Bucci F. Daily variation of brain-derived neurotrophic factor and cortisol in women with normal menstrual cycles, undergoing oral contraception and in postmenopause. Hum Reprod. (2009) 24:2303–9. 10.1093/humrep/dep119 19491202

[17] Hoehner JC Wester T Pahlman S Olsen L. Localization of neurotrophins and their high-affinity receptors during human enteric nervous system development. Gastroenterology. (1996) 110:756–67. 10.1053/gast.1996.v110.pm8608885 8608885

[18] Sen S Duman R Sanacora G. Serum brain-derived neurotrophic factor, depression, and antidepressant medications: meta-analyses and implications. Biol Psychiatry. (2008) 64:527–32. 10.1016/j.biopsych.2008.05.005 18571629 PMC2597158

[19] Yu YB Zuo XL Zhao QJ Chen FX Yang J Dong YY. Brain-derived neurotrophic factor contributes to abdominal pain in irritable bowel syndrome. Gut. (2012) 61:685–94. 10.1136/gutjnl-2011-300265 21997550

[20] Wang P Du C Chen FX Li CQ Yu YB Han T. BDNF contributes to IBS-like colonic hypersensitivity via activating the enteroglia-nerve unit. Sci Rep. (2016) 6:20320. 10.1038/srep20320 26837784 PMC4738267

[21] Zhang Y Qin G Liu DR Wang Y Yao SK. Increased expression of brain-derived neurotrophic factor is correlated with visceral hypersensitivity in patients with diarrhea-predominant irritable bowel syndrome. World J Gastroenterol. (2019) 25:269–81. 10.3748/wjg.v25.i2.269 30670915 PMC6337018

[22] Wang P Chen FX Du C Li CQ Yu YB Zuo XL. Increased production of BDNF in colonic epithelial cells induced by fecal supernatants from diarrheic IBS patients. Sci Rep. (2015) 5:10121. 10.1038/srep10121 25998025 PMC4441152

[23] Coulie B Szarka LA Camilleri M Burton DD McKinzie S Stambler N. Recombinant human neurotrophic factors accelerate colonic transit and relieve constipation in humans. Gastroenterology. (2000) 119:41–50. 10.1053/gast.2000.8553 10889153

[24] Mahurkar-Joshi S Videlock EJ Iliopoulos D Pothoulakis C Mayer EA Chang L 1090 - epigenetic changes in blood cells and colonic mucosa are associated with irritable bowel syndrome (IBS). Gastroenterology. (2018) 154:S-214. 10.1016/S0016-5085(18)31105-3

[25] Videlock EJ Mahurkar-Joshi S Hoffman JM Iliopoulos D Pothoulakis C Mayer EA. Sigmoid colon mucosal gene expression supports alterations of neuronal signaling in irritable bowel syndrome with constipation. Am J Physiol Gastrointest Liver Physiol. (2018) 315:G140–57. 10.1152/ajpgi.00288.2017 29565640 PMC6109711

[26] Longstreth GF Thompson WG Chey WD Houghton LA Mearin F Spiller RC. Functional bowel disorders. Gastroenterology. (2006) 130:1480–91. 10.1053/j.gastro.2005.11.061 16678561

[27] Francis CY Morris J Whorwell PJ. The irritable bowel severity scoring system: a simple method of monitoring irritable bowel syndrome and its progress. Aliment Pharmacol Ther. (1997) 11:395–402. 10.1046/j.1365-2036.1997.142318000.x 9146781

[28] Vandesompele J De Preter K Pattyn F Poppe B Van Roy N De Paepe A Accurate normalization of real-time quantitative RT-PCR data by geometric averaging of multiple internal control genes. Genome Biol. (2002) 3:RESEARCH0034 10.1186/gb-2002-3-7-research0034 12184808 PMC126239

[29] Riedl A Schmidtmann M Stengel A Goebel M Wisser AS Klapp BF. Somatic comorbidities of irritable bowel syndrome: a systematic analysis. J Psychosom Res. (2008) 64:573–82. 10.1016/j.jpsychores.2008.02.021 18501257

[30] Mowla SJ Farhadi HF Pareek S Atwal JK Morris SJ Seidah NG. Biosynthesis and post-translational processing of the precursor to brain-derived neurotrophic factor. J Biol Chem. (2001) 276:12660–6. 10.1074/jbc.M008104200 11152678

[31] Teng HK Teng KK Lee R Wright S Tevar S Almeida RD. ProBDNF induces neuronal apoptosis via activation of a receptor complex of p75NTR and sortilin. J Neurosci. (2005) 25:5455–63. 10.1523/JNEUROSCI.5123-04.2005 15930396 PMC6724992

[32] Mowla SJ Pareek S Farhadi HF Petrecca K Fawcett JP Seidah NG. Differential sorting of nerve growth factor and brain-derived neurotrophic factor in hippocampal neurons. J Neurosci. (1999) 19:2069–80. 10.1523/JNEUROSCI.19-06-02069.1999 10066260 PMC6782557

[33] Osler W The Principles and Practice of Medicine. New York, NY: Appleton & Co. (1892).

[34] Macias M Dwornik A Ziemlinska E Fehr S Schachner M Czarkowska-Bauch J. Locomotor exercise alters expression of pro-brain-derived neurotrophic factor, brain-derived neurotrophic factor and its receptor TrkB in the spinal cord of adult rats. Eur J Neurosci. (2007) 25:2425–44. 10.1111/j.1460-9568.2007.05498.x 17445239

[35] Lu B Pang PT Woo NH. The yin and yang of neurotrophin action. Nat Rev Neurosci. (2005) 6:603–14. 10.1038/nrn1726 16062169

[36] Lee R Kermani P Teng KK Hempstead BL. Regulation of cell survival by secreted proneurotrophins. Science. (2001) 294:1945–8. 10.1126/science.1065057 11729324

[37] Egan MF Kojima M Callicott JH Goldberg TE Kolachana BS Bertolino A. The BDNF val66met polymorphism affects activity-dependent secretion of BDNF and human memory and hippocampal function. Cell. (2003) 112:257–69. 10.1016/S0092-8674(03)00035-7 12553913

[38] Bathina S Das UN. Brain-derived neurotrophic factor and its clinical implications. Arch Med Sci. (2015) 11:1164–78. 10.5114/aoms.2015.56342 26788077 PMC4697050

[39] Fu Y Lin YM Winston JH Radhakrishnan R Huang LM Shi XZ. Role of brain-derived neurotrophic factor in the pathogenesis of distention-associated abdominal pain in bowel obstruction. Neurogastroenterol Motil. (2018) 30:e13373. 10.1111/nmo.13373 29781158 PMC6160336

[40] Leung PS The Gastrointestinal System - Gastrointestinal, Nutritional and Hepatobiliary Physiology. Dordrecht; Heidelberg; New York, NY; London: Springer (2014).

[41] Sarna SK. Enteric descending and afferent neural signaling stimulated by giant migrating contractions: essential contributing factors to visceral pain. Am J Physiol Gastrointest Liver Physiol. (2007) 292:G572–81. 10.1152/ajpgi.00332.2006 16990445

[42] Xu XJ Liang L Yao SK. Nerve growth factor and diarrhea-predominant irritable bowel syndrome (IBS-D): a potential therapeutic target? Biomed Biotechnol. (2016) 17:1–9. 10.1631/jzus.B1500181 26739521 PMC4710835

[43] Hungin AP Whorwell PJ Tack J Mearin F. The prevalence, patterns and impact of irritable bowel syndrome: an international survey of 40,000 subjects. Aliment Pharmacol Ther. (2003) 17:643–50. 10.1046/j.1365-2036.2003.01456.x 12641512

[44] Jung HK Halder S McNally M Locke GR III Schleck CD Zinsmeister AR. Overlap of gastro-oesophageal reflux disease and irritable bowel syndrome: prevalence and risk factors in the general population. Aliment Pharmacol Ther. (2007) 26:453–61. 10.1111/j.1365-2036.2007.03366.x 17635380

[45] Numakawa T Yokomaku D Richards M Hori H Adachi N Kunugi H. Functional interactions between steroid hormones and neurotrophin BDNF. World J Biol Chem. (2010) 1:133–43. 10.4331/wjbc.v1.i5.133 21540998 PMC3083963

[46] Karisetty BC Joshi PC Kumar A Chakravarty S. Sex differences in the effect of chronic mild stress on mouse prefrontal cortical BDNF levels: a role of major ovarian hormones. Neuroscience. (2017) 356:89–101. 10.1016/j.neuroscience.2017.05.020 28527954

[47] Pan XQ Malykhina AP. Estrous cycle dependent fluctuations of regulatory neuropeptides in the lower urinary tract of female rats upon colon-bladder cross-sensitization. PLoS ONE. (2014) 9:e94872. 10.1371/journal.pone.0094872 24788240 PMC4006778

[48] Canavan C West J Card T. The epidemiology of irritable bowel syndrome. Clin Epidemiol. (2014) 6:71–80. 10.2147/CLEP.S40245 24523597 PMC3921083

[49] Choi YK Kraft N Zimmerman B Jackson M Rao SS. Fructose intolerance in IBS and utility of fructose-restricted diet. J Clin Gastroenterol. (2008) 42:233–8. 10.1097/MCG.0b013e31802cbc2f 18223504

[50] Jung KW Seo M Cho YH Park YO Yoon SY Lee J. Prevalence of fructose malabsorption in patients with irritable bowel syndrome after excluding small intestinal bacterial overgrowth. J Neurogastroenterol Motil. (2018) 24:307–16. 10.5056/jnm17044 29433301 PMC5885730

[51] Ohman L Simren M. Pathogenesis of IBS: role of inflammation, immunity and neuroimmune interactions. Nat Rev Gastroenterol Hepatol. (2010) 7:163–73. 10.1038/nrgastro.2010.4 20101257

[52] Kennedy PJ Clarke G Quigley EM Groeger JA Dinan TG Cryan JF. Gut memories: towards a cognitive neurobiology of irritable bowel syndrome. Neurosci Biobehav Rev. (2012) 36:310–40. 10.1016/j.neubiorev.2011.07.001 21777613

[53] Johlin FC Jr Panther M Kraft N. Dietary fructose intolerance: diet modification can impact self-rated health and symptom control. Nutr Clin Care. (2004) 7:92–7. 15624540

[54] Yang J Deng Y Chu H Cong Y Zhao J Pohl D. Prevalence and presentation of lactose intolerance and effects on dairy product intake in healthy subjects and patients with irritable bowel syndrome. Clin Gastroenterol Hepatol. (2013) 11:262–8 e261. 10.1016/j.cgh.2012.11.034 23246646

