# Supplementary figures and images for: The Role of Brain-Derived Neurotrophic Factor in Irritable Bowel Syndrome
Source: Front Psychiatry. 2021 Jan 14;11:531385. doi: 10.3389/fpsyt.2020.531385 (PMC7840690; doi:10.3389/fpsyt.2020.531385)

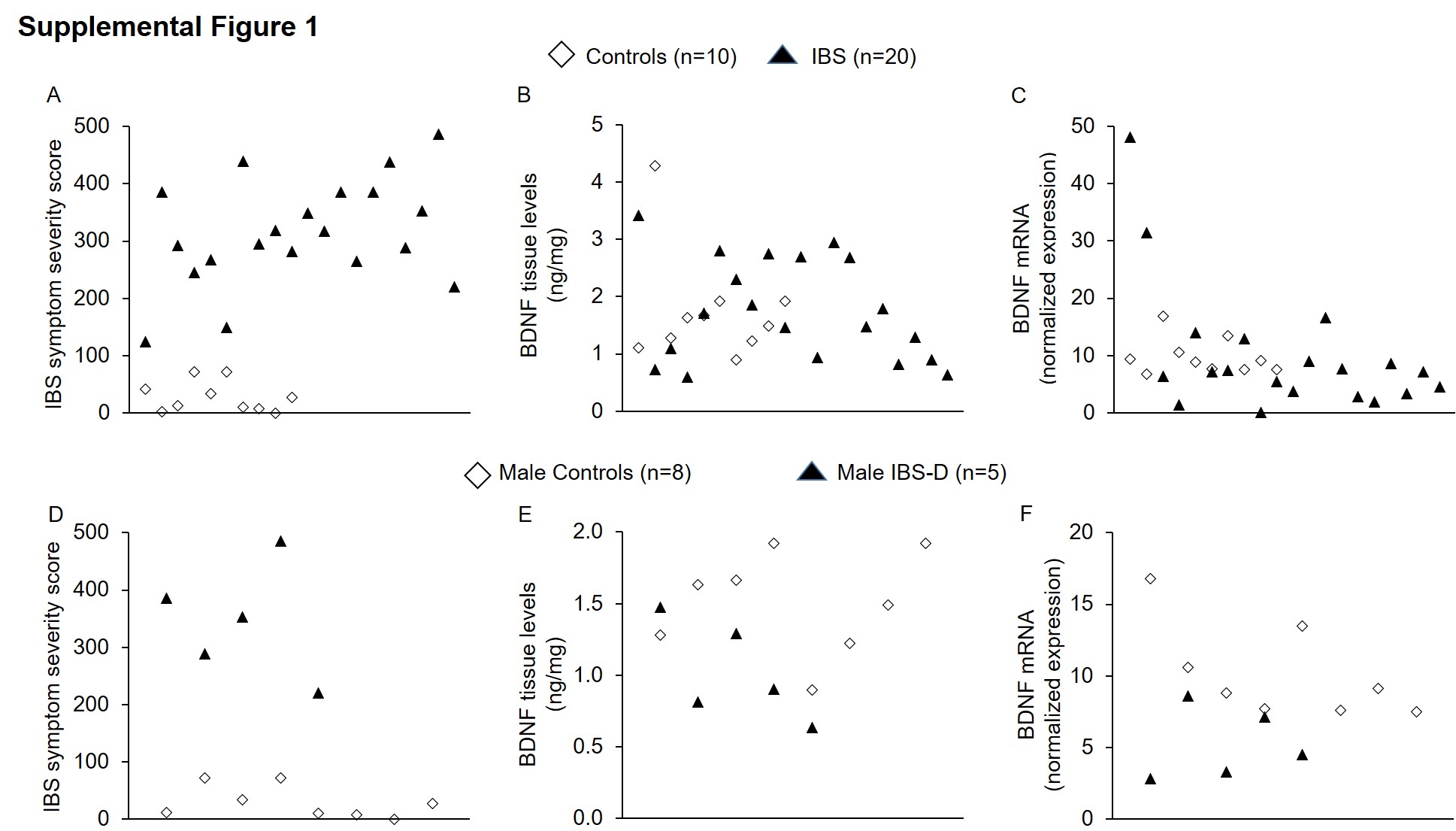

Supplement: Supplementary Figure 1 — Graphs are based on pooled data shown in Figure 2. The Supplementary Figure additionally shows individual data values. Details and levels of significance are displayed in Figure 2. (A) IBS symptom severity in healthy controls vs. IBS patients. (B) Measurement of BDNF protein tissue levels obtained from colonic biopsies of the descending colon by ELISA in healthy controls and IBS patients. (C) Measurement of BDNF mRNA levels obtained from colonic biopsies of the descending colon by ELISA in healthy controls and IBS patients. (D) IBS symptom severity in healthy male controls vs. male IBS-D patients. (E) Measurement of BDNF protein tissue levels obtained from colonic biopsies of the descending colon by ELISA in healthy male controls and male IBS-D patients. (F) Measurement of BDNF mRNA levels obtained from colonic biopsies of the descending colon by ELISA in healthy male controls and male IBS-D patients. [file Image_1.JPEG]
