# Supplementary material for: The Role of Brain-Derived Neurotrophic Factor in Irritable Bowel Syndrome
Source: Front Psychiatry. 2021 Jan 14;11:531385. doi: 10.3389/fpsyt.2020.531385 (PMC7840690; doi:10.3389/fpsyt.2020.531385)
Supplement: Supplementary Table 1 — IBS symptom severity scores and BDNF levels in IBS-D subgroup. [file Data_Sheet_1.PDF]

Supplemental table 1. IBS Symptom Severity Scores and BDNF levels in IBS-D subgroup

**Parameter**	**Women**			**Men**		
	**Control** (n=2)	**IBS-D** (n=9)	***p***	**Control** (n=8)	**IBS-D** (n=5)	***p***
IBS-SSS	21.5 ± 19.5 (2 – 41)	270.0 ± 17.1 (149 – 319)	n.c.	29.3 ± 10.1 (0 – 72)	346.6 ± 45.0 (149 – 319)	**<0.001**
BDNF mRNA (arbitrary unit)	8.1 ± 1.4 (6.7 – 9.4)	8.0 ± 1.4 (1.4 – 12.9)	n.c.	10.2 ± 1.2 (7.5 – 16.8)	5.3 ± 1.1 (1.4 – 12.9)	**0.016**
BDNF protein (ng/mg)	2.7 ± 1.6 (1.1 – 4.3)	1.8 ± 0.3 (0.6 – 2.8)	n.c.	1.5 ± 0.1 (0.9 – 1.9)	1.0 ± 0.2 (0.6 – 2.8)	**0.036**

BDNF levels were assessed using nCounter analysis and ELISA and corrected for housekeeping gene expression or total tissue protein, respectively. Data are presented as mean ± sem, the range is indicated in parentheses. Normality was assessed using the Kolmogorow-Smirnov test. Since data were normally distributed, differences were assessed by t-test. Significant differences are displayed in bold. Abbreviations: BDNF, brain-derived neurotrophic factor; IBS-D, diarrhea-predominant irritable bowel syndrome, IBS-SSS, IBS Symptom Severity Score; n.c. not calculated.

Supplemental table 2. IBS-SSS and BDNF levels in IBS patients without fructose malabsorption (IBS-FM) or with (IBS+FM)

**Parameter**	**IBS-FM** (n=10)	**IBS+FM** (n=10)	***p***
IBS-SSS	316.2 ± 31.4 (124 - 438)	312.1 ± 28.6 (149 – 486)	0.575
BDNF mRNA (arbitrary unit)	10.2 ± 4.4 (1.4 – 48.1)	10.8 ± 2.8 (2.8 – 31.5)	0.918
BDNF protein (ng/mg)	1.8 ± 0.3 (0.6 – 3.4)	1.7 ± 0.3 (0.7 – 2.8)	0.904

BDNF levels were assessed using nCounter analysis and ELISA and corrected for housekeeping gene expression or total tissue protein, respectively. Data are presented as mean ± sem, the range is indicated in parentheses. Normality was assessed using the Kolmogorow-Smirnov test. Since data were normally distributed, differences were assessed by t-test. No significant differences were found. Abbreviations: BDNF, brain-derived neurotrophic factor; FM, fructose malabsorption; IBS, irritable bowel syndrome, IBS-SSS, IBS Symptom Severity Score.
